# Human Tuberculosis Caused by *Mycobacterium bovis*, Taiwan

**DOI:** 10.3201/eid1403.070058

**Published:** 2008-03

**Authors:** Ruwen Jou, Wei-Lun Huang, Chen-Yuan Chiang

**Affiliations:** * Centers for Disease Control, Taipei, Taiwan, Republic of China; † International Union Against Tuberculosis and Lung Disease, Paris, France

**Keywords:** Mycobacterium bovis, tuberculosis, human cases, Taiwan, letter

## Abstract

Human Tuberculosis Caused by *Mycobacterium bovis*, Taiwan

**To the Editor:** *Mycobacterium bovis* is one of the causative agents of tuberculosis (TB) in humans and animals. Drinking unpasteurized milk, eating undercooked meat, and close contact with infected animals are the main sources of infection for humans. Currently, 119 *M*. *bovis* spoligotypes are contained in the fourth international spoligotyping database (SpolDB 4) and are categorized into 3 main sublineages corresponding to ST prototypes 482, 683, and 479 (*1*).

Although an *M*. *bovis* surveillance program for farm animals has been implemented by the Taiwan Council of Agriculture, no surveillance system exists for human TB cases caused by *M*. *bovis*. To monitor the epidemiology of *M*. *bovis* in domestic animals, a regular tuberculin skin test (TST) is compulsory for cattle and sheep and optional for deer in Taiwan (*2*). In 2005, screening of *Mycobacterium* spp. infections by TST was performed for 111,412 cattle and 73,396 caprint and ovine herds, of which 188 (0.17%) and 148 (0.2%), respectively, were positive (*2*). We used spacer oligonucleotide typing (spoligotyping) and mycobacterial interspersed repetitive units–variable number tandem repeat (MIRU-VNTR) methods to investigate human TB caused by *M*. *bovis* in Taiwan.

During 2004–2005, a total of 3,321 mycobacteria isolates from individual patients were sent to the reference laboratory for strain typing. Of the 3,321 patients, 2,427 (73.1%) were male, 903 (27.2%) were from eastern Taiwan, and 513 (15.4%) were aboriginal persons. The mean age of the patients was 58.7 years: 224 (6.7%) were <25 years of age, 667 (20.1%) were 25–44 years of age, 906 (27.3%) were 45–64 years of age, and 1,524 (45.9%) were >65 years of age.

Isolates were screened by using a GenoType kit (*3*) and multiplex PCR (*4*). To differentiate between *M*. *bovis* and *M*. *bovis* BCG strains, presence or absence of region of difference 1 was analyzed (*5*). Spoligotyping was performed with a commercial kit (Isogen Bioscience BV, Maarssen, the Netherlands) following the manufacturer’s instructions (*6*). Spoligotyping profiles were analyzed by using Bionumerics software, version 4.51 (Applied Maths, Kortijk, Belgium). The resolved spoligotype was designated by comparing it to SpolDB4 (*1*). The MIRU-VNTR assay was performed by using a modified, high-throughput, 15-loci MIRU typing system that we developed (*7*).

Of the 3,321 patient isolates, 3,306 (99.5%) were *M*. *tuberculosis* and only 15 (0.5%) were *M*. *bovis*. Mean age of the 15 *M*. *bovis*–infected patients was 62.2 years (Table). Twelve (80%) patients were male, and 3 (20%) were female. Of these 15 patients, 10 (66.7%) had newly diagnosed TB and 5 (33.3%) had been treated with anti-TB drugs. Most (11/15, 73%) of the patients were from eastern Taiwan, where the reporting rate for TB was highest among the 4 regions of Taiwan; 60% (9/15) were aboriginal persons. Of the 15 patients, 13 (86.7%) had pulmonary TB, 1 had both pulmonary and extrapulmonary TB, and 1 had extrapulmonary TB. Only 2 patients (cases 2 and 3) had known contact with farm animals. Univariate analysis showed that region (eastern region 1.2% vs. other region 0.2%; odds ratio [OR] 7.4, 95% confidence interval [CI] (2.4–23.4) and ethnicity (aboriginal 1.8% vs. nonaboriginal 0.2%; OR 8.3, 95% CI 3.0–23.5) were associated with *M*. *bovis* infection but not age and sex. The association between region and *M*. *bovis* disappeared after controlling for age and ethnicity. Aboriginal ethnicity was the only factor significantly associated with TB caused by *M*. *bovis* after controlling for age (adjusted OR 12.7, 95% CI 4.2–38.9).

**Table:** Characteristics of 15 patients with tuberculosis caused by *Mycobacterium bovis*, Taiwan, 2004–2005

Patient no.	Age at diagnosis, y	Sex	Type of tuberculosis	Ethnicity	Region
1	53	M	Pulmonary	Aboriginal	Northern
2	71	M	Pulmonary	Han	Central
3	76	M	Pulmonary	Han	Central
4	84	M	Pulmonary	Han	Southern
5	71	F	Pulmonary	Aboriginal	Eastern
6	55	M	Pulmonary	Aboriginal	Eastern
7	51	M	Pulmonary	Aboriginal	Eastern
8	65	F	Pulmonary	Aboriginal	Eastern
9	51	M	Pulmonary	Han	Eastern
10	90	M	Pulmonary	Han	Eastern
11	34	M	Pulmonary	Aboriginal	Eastern
12	81	M	Pulmonary	Han	Eastern
13	64	F	Pulmonary	Aboriginal	Eastern
14	44	M	Bladder/urinary	Aboriginal	Eastern
15	43	M	Pulmonary/miliary	Aboriginal	Eastern

Spoligotyping profiles of the 15 *M*. *bovis* isolates were typical of *M*. *bovis* with the absence of spacers 3, 6, 9, 16, 21, and 39–43 (*8*). Although 15 cases were reported separately from different regions of Taiwan, only 1 spoligotyping profile was identical to spoligotype ST 684 of the bovis sublineage. In addition, we identified 2 similar MIRU-VNTR profiles: 523–23232–42533–22 (13/14, 92.9%, cases 1–9, 11–13, and 15) and 523–22232–42523–22 (1/14, 7.1%, case 14). We were unable to obtain sufficient DNA from 1 strain (case 10) for MIRU-VNTR typing.

Aboriginal persons in Taiwan were more likely to have TB caused by *M*. *bovis* and had a 5-fold higher reporting rate for TB (*9*) than nonaboriginal persons. Environmental and genetic factors may be associated with a higher reporting rate for TB among aboriginal populations (*10*), but the contribution of *M*. *bovis* infection needs to be investigated. Because only 1 major spoligotype and 2 similar MIRU patterns were found in this case series, spread of a predominant clone in Taiwan is likely. In addition, because of insufficient epidemiologic data, we were unable to determine the proportion of cases caused by reactivation of latent infection and those caused by recent transmission. In our study population, *M*. *bovis* infection in humans appeared to be predominately indigenous in Taiwan because no imported case was noted. We are now genotyping *M*. *bovis* strains isolated from farm animals to help elucidate the source of infection and transmission of *M*. *bovis* in Taiwan.
